# Successful treatment of gynecologic malignancy with trastuzumab deruxtecan in three patients with *HER2* mutations

**DOI:** 10.1016/j.gore.2025.101974

**Published:** 2025-10-13

**Authors:** Divya S. Choudhury, Katherine C. Kurnit, S. Diane Yamada, Nita K. Lee, Anusha Vemuri, Julieta E. Barroeta, Shayan Rayani, Christopher M. Straus, Gini F. Fleming, Josephine S. Kim

**Affiliations:** a Pritzker School of Medicine, University of Chicago, Chicago, IL, USA; b Department of Obstetrics and Gynecology, Section of Gynecologic Oncology, University of Chicago Medical Center, Chicago, IL, USA; c Department of Pathology, University of Chicago Medical Center, Chicago, IL, USA; d Department of Medicine, Section of Hematology/Oncology, University of Chicago Medical Center, Chicago, IL, USA; e Department of Radiology, University of Chicago Medical Center, Chicago, IL, USA

## Abstract

**Objectives:**

Trastuzumab deruxtecan is an antibody-drug conjugate that was recently FDA-approved as second-line therapy for advanced solid tumors expressing HER2 by immunohistochemistry at the 3+ level. Clinical trials have demonstrated trastuzumab deruxtecan’s promise as a treatment for advanced gynecologic malignancies, which often have poor prognoses and limited treatment options. Here, we present three HER2 negative (IHC 0) or untested patients with *HER2*-mutated gynecologic cancers who derived significant benefit from trastuzumab deruxtecan.

**Methods:**

Consent from patients to carry out this research was obtained, and their cases were reviewed via the electronic medical record. Relevant clinical, surgical, pathologic, and imaging data are described in this report.

**Results:**

These patients with *HER2-*mutated disease benefited from trastuzumab deruxtecan despite being HER2 negative (IHC 0) or untested by immunohistochemistry and therefore not meeting current criteria for FDA-approved treatment with this drug. The first case describes a 65-year-old woman with recurrent high grade endometrial carcinoma who had a progression-free survival of over 12 months with trastuzumab deruxtecan. In the second case, a 76-year-old woman with recurrent low grade serous ovarian carcinoma received trastuzumab deruxtecan for over two years and continues the treatment at the time of this article’s submission. The third case describes a 52-year-old woman with recurrent cervical mesonephric adenocarcinoma who had a 15-month long partial response to trastuzumab deruxtecan.

**Conclusions:**

These cases illustrate the potential for trastuzumab deruxtecan to be useful among a broader range of patients than current guidelines include and suggest that comprehensive genomic testing is necessary to help identify patients whose disease may be susceptible to HER2-targeted therapies.

## Highlights

• Trastuzumab deruxtecan, which was FDA-approved in 2023, shows particular promise in HER2-expressing gynecologic malignancies.
• This report shows trastuzumab deruxtecan’s efficacy among three patients with *HER2* mutations rather than HER2 overexpression.
• *HER2*-mutational testing can help identify patients who could benefit from trastuzumab deruxtecan.

## 1 Introduction

Metastatic gynecologic malignancies have poor prognoses with limited Food & Drug Administration (FDA)-approved treatment options. Antibody-drug conjugates are an exciting class of drugs with substantial applicability in multiple gynecologic malignancies. One example is trastuzumab deruxtecan (T-DXd), which was approved in August 2023 as second line therapy for any advanced solid tumor expressing HER2 (also known as ERBB2) by immunohistochemistry (IHC) at the 3+, or maximal, level. The trastuzumab component of T-DXd binds the HER2 receptor expressed by the tumor cells and the receptor-drug complex is subsequently endocytosed. Deruxtecan, a topoisomerase I inhibitor, is linked to the antibody and exerts a cytotoxic effect on the target cell. T-DXd has also been shown to have a cytotoxic effect on proximate non-HER2-expressing cells in the tumor microenvironment, possibly due to the membrane permeability of deruxtecan and/or the presence of cathepsins in the tumor microenvironment that catalyze extracellular linker cleavage (Shiraishi et al., 2025, Kepp and Kroemer, 2025).

Efficacy leading to FDA approval of T-DXd was demonstrated in the DESTINY-PanTumor02 Phase II trial (Meric-Bernstam et al., 2024). The trial enrolled patients whose tumors expressed HER2 by IHC at the 1+, 2+, or 3+ level. The published data from this study included 40 patients with endometrial cancer, 40 patients with cervical cancer, and 40 patients with ovarian cancer, all with either 2+ or 3+ expression. The objective response rate (ORR) was 57.5% for endometrial cancer patients, 50.0% for cervical cancer patients, and 45.0% for ovarian cancer patients. The response was more robust in patients with HER2 IHC 3+ as compared with 2+ tumors. Across all solid tumors included, the ORR was 51.4% for patients with IHC 3+ tumors and 26.5% for those with IHC 2+ tumors (Oaknin et al., 2024). Median duration of response was not reached in the endometrial cancer cohort, 14.2 months in the cervical cancer cohort, and 11.3 months in the ovarian cancer cohort. Across all studied tumor types, the magnitude of benefit was greatest among patients with gynecologic cancers. T-DXd has also shown promise for gynecologic malignancies in additional clinical trials. For example, the STATICE trial demonstrated efficacy of T-DXd in patients with advanced or recurrent endometrial carcinosarcoma with HER2 IHC scores of 1+, 2+, or 3+ (Nishikawa et al., 2023). These results suggest a potential role for T-DXd in this patient population with broader HER2 expression.

HER2 expression is typically tested using IHC. Expression at the IHC 3+ level is strongly correlated with amplification of the *HER2 (ERBB2)* gene. In high-risk, metastatic, or recurrent endometrial cancer, recently reported rates of HER2 overexpression by IHC were 17.2% at the 1+ or 2+ level and 7.9% at the 3+ level (van Dijk et al., 2025). In a broader population-based series, however, the frequency of HER2 “positive” IHC using criteria of ≥1+ was as high as 87% (Krakstad et al., 2024). Another study analyzing 2,042 endometrial cancers found *HER2* amplification by next generation sequencing (NGS) in 3.8% of endometrial cancer across subtypes, including 8% of serous carcinomas, 7.1% of carcinosarcomas, 6% of clear cell carcinomas, and 0.2% of endometrioid carcinomas (Ross et al., 2022). This study also notes that HER2 amplification was more strongly associated with high grade histology and *TP53* mutation than with tumor cell type.

Further complexity in identifying the appropriate patient population for HER2-directed therapy arises from the fact that HER2 expression may vary among biopsies taken from a single patient over time. One study found heterogeneity of HER2 expression in endometrial cancer, with a loss of HER2 expression in metastatic lesions compared to primary tumor (Halle et al., 2018). This data suggests that the tumor genetic and molecular profile determined from a single biopsy specimen may not generalize to the patient’s entire disease spectrum and implies a potential role for repeat testing of metastatic lesions in the setting of recurrent disease.

Although HER2 overactivity can be due to *HER2* amplification, it can also result from *HER2* activating mutations. *HER2* activating mutations are much less common and are not always associated with IHC positivity. Pathogenic *HER2* mutations, detected using NGS, are seen in about 3.5% of tumors across cancer types (Pahuja et al., 2018), including 2.6% of endometrial cancers (Brodeur et al., 2024). T-DXd is FDA-approved for patients with lung cancer whose tumors have *HER2* mutations with reports showing an overall response rate of 58% (Mehta et al., 2024). The DESTINY-PanTumor01 trial included patients based on NGS- or PCR-detected presence of prespecified activating *HER2* mutations (and excluded any tumor expressing HER2 at the 3+ level by IHC) and showed an ORR of 29.4% across tumor types (Li et al., 2024). However, only six patients with gynecologic malignancies were included, and T-DXd is not FDA-approved for gynecologic malignancies based on *HER2* gene mutations at this time.

We present three patients with *HER2* mutations—one with low grade serous ovarian cancer, one with endometrioid endometrial cancer, and one with cervical mesonephric adenocarcinoma—who responded well to T-DXd. This case series was IRB-approved, and all three patients signed written informed consent for publication.

## 2 Clinical cases

### 2.1 Case 1

A 65-year-old woman was diagnosed with recurrent high grade endometrial carcinoma with serous and endometrioid features. Initial surgery showed disease limited to the uterus and endocervical canal, however subsequent CT imaging showed inguinal adenopathy consistent with stage IV disease. She received paclitaxel and carboplatin and had a complete response by imaging. Immunohistochemical stains demonstrated patchy ER and PR, p53 overexpression, and retained mismatch repair proteins. HER2 immunohistochemical stain was negative (0+), however NGS showed a pathogenic mutation in *HER2* p.G776V. Eighteen months later, imaging showed multiple pelvic and retroperitoneal lymph nodes as well as omental and peritoneal lesions consistent with metastatic disease. She then had a partial response to checkpoint inhibitor-based therapy on a clinical trial before having further disease progression after eight months. Paclitaxel and carboplatin were re-administered over three months with a partial response to treatment followed by two additional lines of cytotoxic chemotherapy, including bevacizumab, with progression.

**Fig. 1:**
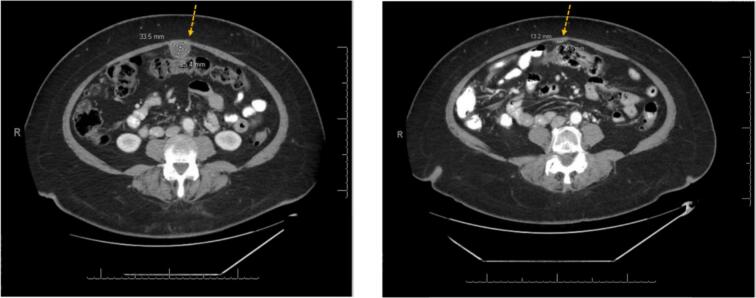
**CT imaging demonstrates response to treatment with T-DXd in Case 1.** Focal metastatic lesion decreases in size from 33.5 mm x 25.4 mm (left) to 13.2 mm to 5.5 mm (right) with T-DXd treatment over the course of six months.

**Fig. 2:**
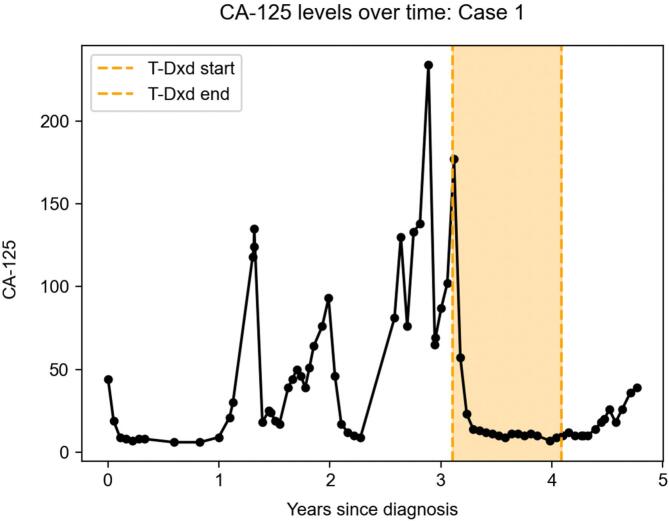
**CA-125 levels illustrate response to T-DXd in Case 1.** CA-125 level fell from 177 to 23 after initiation of T-DXd and remained low while the patient received the drug.

In light of the activating *HER2* mutation, the patient then began T-DXd. Her CA-125 decreased from 177 to 23 within six weeks of treatment, and CT imaging showed decreasing size of metastatic lesions (Fig. 1). She received 12 additional months of T-DXd with imaging showing stable, low-volume disease and CA-125 levels in the normal range (range 7–14) (Fig. 2). After cycle 17, T-DXd was held due to thrombocytopenia and hyperbilirubinemia, and a bone marrow biopsy revealed myelodysplastic syndrome (MDS). CT imaging five months later showed progression of her endometrial cancer. The patient began single agent trastuzumab to minimize hematologic side effects. As of five months on treatment, she remains on this therapy with stable disease. The total duration of this patient’s treatment from diagnosis of metastatic disease to present is four years and five months.

### 2.2 Case 2

A 76-year-old woman was diagnosed with recurrent low grade serous ovarian carcinoma with unresectable disease seen on diagnostic laparoscopy. Initially, she received two cycles of paclitaxel and carboplatin followed by two cycles of gemcitabine and carboplatin resulting in a partial response. The patient received letrozole resulting in stable disease for one year. She then underwent radical cytoreductive surgery with completely resected peritoneal disease and a residual 2 cm mass inside the cecum. Colonoscopy later confirmed multifocal low grade serous carcinoma inside the colon. She resumed letrozole for the next 12 months at which point her CA-125 level started to rise and a repeat colonoscopy showed possible progression. She then began oral cyclophosphamide and bevacizumab with disease progression after 6 months.

NGS revealed a large deletion of *HER2,* including the entirety of exon 16 and a portion of intron 16 (*HER2* c.1900_1946 + 854del). Deletion of exon 16 leads to oncogenic activation of the HER2 protein (Turpin et al., 2016). Approximately 5% of low-grade serous ovarian carcinomas harbor activating *HER2* mutations (Neil et al., 2025). HER2 immunohistochemical stain performed on a small metastatic deposit was negative (IHC 0). Because of the activating *HER2* deletion, the patient began single agent trastuzumab and her disease remained stable for over two years. She was then treated on a clinical trial targeting the MAPK pathway for eight months when her cancer progressed again.

**Fig. 3:**
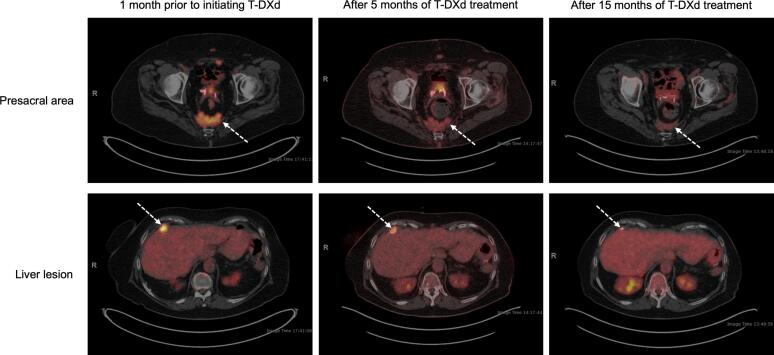
**PET imaging demonstrates response to treatment with T-DXd in Case 2.** Top row: Metastatic disease in the presacral area with decreasing avidity on PET imaging demonstrates patient’s response to treatment with T-DXd over time. Bottom row: Liver lesion with decreasing avidity and size after five and 15 months of T-DXd treatment also shows response to treatment.

At this time, based on the initial *HER2* mutation and excellent prior biochemical response to trastuzumab, the patient began treatment with T-DXd. Initial follow-up imaging showed a partial response with ongoing improvement in avidity on serial PET scans (Fig. 3). She remains on T-DXd after over two years. After cycle 42, the patient developed neutropenia requiring a decrease in dose frequency from every three weeks to every four weeks.

### 2.3 Case 3

A 52-year-old woman was diagnosed with recurrent cervical mesonephric adenocarcinoma. Her initial diagnosis was a high-grade adenocarcinoma of uterine versus cervical origin, presumptive stage IIB. She was treated with preoperative external beam pelvic radiation for one month followed by robotic-assisted total hysterectomy with bilateral salpingo-oophorectomy, removal of the left parametrium, and pelvic lymph node dissection. Pelvic nodes were negative at the time of surgery, and the patient was cancer free postoperatively. She received three cycles of adjuvant paclitaxel and carboplatin and vaginal brachytherapy before being lost to follow up.

Three years later, the patient presented with abdominal pain and CT imaging showing carcinomatosis. A biopsy was consistent with recurrent cervical mesonephric adenocarcinoma. She was treated with 8 cycles of paclitaxel, carboplatin, bevacizumab, and pembrolizumab with a partial response. She was placed on bevacizumab and pembrolizumab maintenance therapy for a year, at which time surveillance CT showed progression with omental caking and peritoneal carcinomatosis.

NGS revealed an activating *HER2* p.I767M mutation. HER2 immunohistochemical staining was not performed. The patient began treatment with T-DXd and imaging about six weeks later showed a partial response to treatment. This partial response was maintained until disease progression 15 months after starting T-DXd. Side effects of T-DXd included gastrointestinal toxicity and neutropenia, which required dose reductions.

## 3 Discussion

Here we describe three gynecologic cancer patients with *HER2* mutations: two cases with tumor HER2 expression levels of 0 by IHC, and one case with unknown HER2 expression level by IHC who clinically responded well to T-DXd therapy. In these three cases, T-DXd therapy resulted in a progression-free survival that ranged from 12-28 months for patients who had otherwise limited treatment options. Toxicities were consistent with those commonly observed in the DESTINY-PanTumor02 trial (Meric-Bernstam et al., 2024). Of note, the patient in Case 1 developed MDS which was more likely related to having received four prior lines of cytotoxic chemotherapy. To our knowledge, MDS has not been reported as a known toxicity of T-DXd despite inclusion of a substantial number of heavily pretreated patients in clinical trials (Meric-Bernstam et al., 2024, Li et al., 2024).

In these cases, *HER2-*directed therapy with T-DXd was a treatment option because NGS testing was carried out and showed *HER2* mutations. The phase II SUMMIT trial, which explored the use of neratinib—a pan-epidermal growth factor receptor (EGFR) tyrosine kinase inhibitor with activity against HER2—in the setting of *HER2*-mutated, pretreated cervical cancer, also suggests that testing for *HER2* mutations via NGS may have clinical utility (Friedman et al., 2024). The trial included 22 patients, 18 of whom had adenocarcinoma, and reported an ORR of 18.2% (15% of cervical cancer adenocarcinomas harbor *HER2* mutations) and median duration of response of 7.6 months. The DESTINY PanTumor01 trial, which included patients with multiple solid tumor types based on NGS-detected *HER2* mutations, reported an overall response rate of 29.4% but included only six patients with gynecologic cancers (Li et al., 2024).

Two of our patients also had periods of stability or biomarker response on therapy with trastuzumab. A pooled analysis of the efficacy of anti-HER2 drugs in the treatment of patients with *HER2* mutated cancers of any site noted response rates of 60.0% for T-DXd, 31.0% for pyrotinib, 26.0% for neratinib combined with trastuzumab, 25.0% for neratinib combined with fulvestrant, 19.0% for trastuzumab combined with pertuzumab, and 16.0% for neratinib (Zheng et al., 2023). These results suggest T-DXd is the most active among current anti-HER2 options.

Our report contributes additional evidence that T-DXd could be useful among a broader range of patients than current guidelines suggest. These cases also highlight the complexities that arise in the process of identifying disease that may be susceptible to HER2-targeted therapies. While *HER2* mutations in general are uncommon, there are subsets of patients with gynecologic malignancies (e.g. endocervical cancer) in which *HER2* mutations are more prevalent. For this patient population, because *HER2* mutations will not be identified by conventional FISH or IHC testing for HER2, NGS or another method of genetic testing such as circulating tumor DNA is needed.

T-DXd offers an exciting new HER2-targeting treatment option for patients who have advanced gynecologic cancers; *HER2* mutational testing should be considered as part of the molecular workup particularly for patients with limited alternative treatment options.

## 4 Consent

Written informed consent was obtained from the patient for publication of this case report and accompanying images.

## CRediT authorship contribution statement

**Divya S. Choudhury:** Writing – review & editing, Writing – original draft, Visualization, Investigation. **Katherine C. Kurnit:** Writing – review & editing, Validation, Investigation. **S. Diane Yamada:** Writing – review & editing, Validation, Investigation. **Nita K. Lee:** Writing – review & editing, Validation, Investigation. **Anusha Vemuri:** Writing – review & editing. **Julieta E. Barroeta:** Writing – review & editing. **Shayan Rayani:** Investigation. **Christopher M. Straus:** Visualization, Investigation. **Gini F. Fleming:** Writing – review & editing, Validation, Investigation, Conceptualization. **Josephine S. Kim:** Writing – review & editing, Visualization, Validation, Supervision, Project administration, Investigation, Conceptualization.

## Declaration of Competing Interest

The authors declare that they have no known competing financial interests or personal relationships that could have appeared to influence the work reported in this paper.
